# Validation of the Simplified Chinese Psychoeducational Profile Third Edition in Mainland China

**DOI:** 10.1007/s10803-018-3827-z

**Published:** 2018-12-12

**Authors:** Lu Yu, Xiaoqin Zhu, Daniel T. L. Shek, Xiao-Bing Zou, Hong-Zhu Deng, Peter W. H. Au Yeung

**Affiliations:** 1 Department of Applied Social Sciences, The Hong Kong Polytechnic University, Hong Kong, People’s Republic of China; 2 Behavioral Pediatrics Center, The Third Affiliated Hospital of Sun Yat-sen University, Guangzhou, People’s Republic of China; 3 Heep Hong Society, Hong Kong, People’s Republic of China

**Keywords:** Autism spectrum disorder, Simplified Chinese, Psychoeducational Profile Third Edition, Validation, Performance Test, Caregiver Report

## Abstract

The Psychoeducational Profile 3rd Edition (PEP-3) is a comprehensive assessment tool designed for children with autism spectrum disorder (ASD). Although its original English version has been validated, few validation studies have been conducted on translated versions including Chinese ones. Based on 554 Chinese children with ASD and 311 typically developing Chinese children as the control group, this study investigated the psychometric properties of a simplified Chinese PEP-3 (sCPEP-3) in China mainland. Psychometric evaluation of the sCPEP-3 showed satisfactory internal consistency, test–retest reliability, inter-rater reliability, convergent validity, construct validity, and factorial validity. The findings have several implications such as utilizing the sCPEP-3 in mainland China for customized educational program planning, early identification, and evaluating the treatment effects for children with ASD.

**Electronic supplementary material:**

The online version of this article (10.1007/s10803-018-3827-z) contains supplementary material, which is available to authorized users.

## Introduction

Past decades have witnessed an increasing interest in research on autism spectrum disorder (ASD). A key factor behind this research interest in ASD is its dramatically growing prevalence. One review study has suggested a median prevalence estimate of global ASD of 0.62% (Elsabbagh and Johnson 2010) and a recent survey estimated that ASD occurrence is as high as one in 68 among 8-year-old children in the United States (Centers for Disease Control & Prevention 2014). Children with ASD show symptoms from early childhood, which affect their daily activities and impose a huge burden on their family, community, and wider society (Ganz 2007; Knapp et al. 2007).

Although no medications are currently available with which to cure ASD, the literature suggests that early intervention can ameliorate the prognosis of ASD and improve the functioning of children with ASD in regard to different developmental dimensions, including cognitive ability and language and social skills. For example, early intensive behavioral interventions have been regarded as a “well-established” treatment for ASD (Rogers and Vismara 2008). Evidence even suggests that individuals with ASD should receive behavioral interventions as early as possible because younger participants benefit more from increased behavioral treatment hours than older participants (Granpeesheh et al. 2009). A recent review also concluded that early interventions for children with ASD when they were less than 24 months old not only effectively promote children’s development and social-communicative skills, but also increase parental acceptability and satisfaction (Bradshaw et al. 2015).

To design and implement effective early intervention treatments, one critical step is to assess children’s developmental strengths and deficits in a comprehensive and accurate manner. An ideal assessment not only helps professionals to develop tailored intervention programs that meet the individual needs of children with ASD, but also assists implementers in monitoring children’s development and evaluating the intervention’s effectiveness (Gould et al. 2011). One well recognized assessment tool for children with ASD is the psychoeducational profile (PEP) developed by Schopler and Reichler (1979). The PEP has several unique features (Coonrod and Marcus 2013). First, the PEP evaluates the characteristics of children with ASD from preschool age to elementary age (i.e., 2–7 years old). Second, different from other diagnostic tools (e.g., the Childhood Autism Rating Scale), the PEP is used primarily as an individualized assessment that involves developmental skills, strengths, deficits, and behaviors. Third, by integrating behavioral and developmental information in one instrument, the PEP emphasizes the connection between the atypical features of children with ASD and their developmental delays. As such, the PEP has been widely adopted by practitioners to assess the development of children with ASD over time.

In 1990 and 2005, the PEP was further revised into the PEP-Revised (PEP-R) and then the PEP-third (PEP-3) (Schopler et al. 1990, 2005). While the PEP-R added items corresponding to developmental features for underage children, expanded the language functioning sections, and streamlined items pertaining to problem behaviors, the PEP-3 further updated behavioral items based on latest research findings, utilized more simple, concrete, and interesting materials and instructions, and allowed non-verbal responses. These updates make the PEP-3 suitable for children with ASD aged between 6 months and 7 years old. Furthermore, the PEP-3 added a Caregiver Report to gather information from the child’s caregiver(s) on the child’s self-care skills, behavioral problems, and adaptive behaviors. Given these new features, the PEP-3 can provide a more comprehensive picture for the special learning strengths and weakness, skills, and behaviors of children with ASD, which helps professionals to set up individualized intervention strategies (Schopler et al. 2005). In the test manual, Schopler and colleagues (2005) reported high levels of internal consistency, test–retest reliability, and good criterion-prediction validity of all subtests in the PEP-3, based on a normative sample in the United States.

Given the advances of the PEP-3, it has been utilized in different cultures. However, two limitations should be noted regarding current validation studies on the PEP-3. First, generally speaking, limited validation research has been carried out on the PEP-3 in different cultures. One validation study conducted in Canada (Fulton and D’Entremont 2013) revealed positive correlations between the PEP-3 intellectual and language measures and those measuring similar domains (e.g., the Child Development Inventory) among 136 children with ASD aged between 20 and 75 months. A more recent study (De Giacomo et al. 2016) also detected a positive correlation between PEP-3 cognitive developmental levels and non-verbal IQ, measured by the Leiter International Performance Scale-Revised among 30 Italian children with ASD. However, these studies only focused on some subtests of the PEP-3 and were thus unable to provide comprehensive psychometric evidence for the translated instrument on the whole. Besides, the sample sizes were relatively small, which may also constrain generalizations of the findings.

Second, little is known about the psychometric properties of the PEP-3 in mainland China. While validation studies on the Chinese version of the PEP-3 have been conducted in Taiwan (Fu et al. 2012, 2010) and Hong Kong (Shek and Yu 2014, 2015), validation research on the PEP-3 in mainland China is missing. Based on a small sample (*N* = 63), researchers in Taiwan have investigated the reliability and validity of the Caregiver Report of the Chinese PEP-3, but not the major Performance Test. In contrast, scholars in Hong Kong have conducted systematic validation studies based on a large sample of children with ASD and a comparable number of typically developing children as comparisons (Shek and Yu 2014, 2015). These studies revealed the strong psychometric properties of both the Performance Test and the Caregiver Report of a Cantonese Chinese PEP-3 (CPEP-3). While the evidence strongly supported the usefulness of the CPEP-3 in Hong Kong, it remained unclear whether or not the related findings are applicable to mainland China as well.

This is an important issue that needs to be addressed for three reasons. First, concerning the aforementioned advantages of the PEP-3, it is necessary to validate this questionnaire for its use in mainland China. Second, given that mainland China is the largest Chinese-speaking community in the world, it is indispensable to carry out separate validation research to guarantee the usefulness of the PEP-3 in this area. Third, although Hong Kong and mainland China are both Chinese-speaking regions, they still have significant cultural and language differences, which require different cultural adaptions in developing Chinese versions of research tools.

Specifically, while Cantonese and Traditional Chinese characters are used in Hong Kong, Mandarin and Simplified Chinese characters are employed in most areas of mainland China. Not only are the pronunciations of Cantonese and Mandarin different, there are variations in the vocabulary and grammar of the written Chinese used in the two regions. Currently, the validated CPEP-3 is presented in a manner appropriate for Cantonese-speakers. Therefore, Mandarin users may have difficulty understanding some items and related instructions. Moreover, due to the different societal and political contexts, the lived experiences of children in the two regions are different. For example, the toys, games, and forms of entertainment that are familiar to Hong Kong children are unfamiliar to children living in other Chinese cities. As such, some pictures and objects used in the CPEP-3 need to be adapted. In addition, the developmental norms of children may also be different. To provide an appropriate frame of reference when assessing children with ASD in mainland China, there is a need to develop new CPEP-3 norms based on new samples. In fact, it is a common practice to conduct separate investigations for translated tools that are intended to be used in different Chinese communities (Cen et al. 2017; Gau et al. 2013).

Therefore, to address the related research gaps and provide further evidence for the reliability and validity of translated versions of the PEP-3, the present study investigates the psychometric properties of a Simplified Chinese version of the PEP-3 (sCPEP-3) in mainland China.

## Methods

### Ethical Statement

Prior to the study commencing, approval to conduct the study (including ethical approval) was obtained from the Executive Committee of the Heep Hong Society. The primary caregivers of the participating children gave their written informed consent to the research team. Participants and their primary caregivers were assured that the data collected in the study would be kept confidential.

### Participants and Procedures

The Cantonese Chinese version of the PEP-3 (CPEP-3) was first converted to Simplified Chinese by the Heep Hong Society and reviewed by two bilingual researchers who are native speakers of both Mandarin and Cantonese, including the first author of this paper. Minor modifications were made to the wording of a few items, considering subtle language differences between Cantonese and Mandarin. For example, some words specifically used in Cantonese were replaced by corresponding words in Mandarin. A few more culturally suitable objects were also used to replace the original ones. For example, “church” in the original items was replaced by “park”, as going to church is not a common activity for children in mainland China. The Simplified Chinese version of the PEP-3 (sCPEP-3) was then further examined and compared with the original CPEP-3 by an expert bilingual team to ensure that the two versions were conceptually equivalent.

Two samples of children were recruited to participate in the present study, including a group with ASD and a control group. The group with ASD consisted of 554 children from different areas of China who had been formally diagnosed as having ASD by pediatricians in a well-known child development center in Guangzhou, China. As shown in Table 1, participating children’s ages ranged from 2.0 to 7.9 years, with a male-to-female ratio of 5.30:1 (i.e., 466 boys and 88 girls), which is consistent with the general finding that boys are more frequently diagnosed with ASD (Wang et al. 2016), as girls present different symptoms that are only now beginning to be understood. As compared to the sample recruited for the Hong Kong validation study (Shek and Yu 2015), participants’ age distribution was more even. Particularly, more children with ASD aged between 7.0 and 7.9 years were recruited in the present study.

**Table 1:** Basic demographic characteristics of the ASD group and the control group

Age group (range in years)	2 (2.0–2.9)	3 (3.0–3.9)	4 (4.0–4.9)	5 (5.0–5.9)	6 (6.0–6.9)	7 (7.0–7.9)	Total
ASD group							
No. of participants	76	125	126	104	87	36	554
Percentage	13.7	22.6	22.7	18.8	15.7	6.5	100
No. of girls	16	20	14	19	14	5	88
No. of boys	60	105	112	85	73	31	466
Control group							
No. of participants	53	70	73	61	54	0	311
Percentage	17.0	22.5	23.5	19.6	17.4	0.0	100
No. of girls	7	15	16	14	6	0	58
No. of boys	46	55	57	47	48	0	253

For the control group, 311 children with typical development were recruited from local kindergartens based on the same gender and age ratios as the autistic group. The control group was recruited for the purpose of establishing the construct validity of the sCPEP-3. As shown in Table 1, the children in control group were aged between 2.0 and 6.9 years with a male-to-female ratio of 4.40:1 (i.e., 253 boys and 58 girls).

The sCPEP-3 was first administered on the participating ASD children by trained medical professionals at The Third Affiliated Hospital of Sun Yat-sen University. Before the formal launch of the validation study, these professionals received systematic training from the Heep Hong Society in Hong Kong to ensure that they fully understood the general testing, scoring, and interpreting procedures of the sCPEP-3. Participants with typical development were assessed by the same group of professionals in their respective kindergartens. Parents of children with ASD were also invited to complete the Caregiver Report of the sCPEP-3 on the day during which their children were assessed. No Caregiver Report was administered to parents of children in the control group.

Several subsamples of children with ASD were randomly selected to examine the test–retest reliability, inter-rater reliability, and convergent validity of the sCPEP-3. First, a subsample of 62 autistic children (five girls and 57 boys aged between 2 and 7 years) was assessed twice using the sCPEP-3, with time intervals ranging from 2 weeks to 1 month. Second, inter-rater reliability for the Performance Test of the sCPEP-3 was examined on another subsample of 59 children with ASD who were aged between 2 and 6 years (eight girls and 51 boys). Two experienced researchers independently rated these children on the same day. Third, to examine the convergent validity of sCPEP-3, three established measures that assess developmental problems in children were administered on 60 randomly selected ASD children (55 boys and 5 girls) aged between 2 and 5 years.

### Instruments

#### The Simplified Chinese Version of the Psycho-education Profile-3rd Edition (sCPEP-3)

The sCPEP-3 includes two major components: the Performance Test and the Caregiver Report. The Performance Test consists of ten subtests, including a total of 172 items. The first six subtests measure the child’s developmental level, among which three subtests are designed to measure communication ability: Cognitive Verbal/Preverbal (34 items), Expressive Language (25 items), and Receptive Language (19 items). Another three subtests measure motor ability, including Fine Motor (20 items), Gross Motor (15 items), and Visual-Motor Imitation (10 items) skills. The other four subtests assess maladaptive behaviors, including Affective Expression (11 items), Social Reciprocity (12 items), Characteristic Motor Behaviors (15 items), and Characteristic Verbal Behaviors (11 items). The Caregiver Report has 38 items that are completed by parents or major caregivers based on their daily observations of the child in natural settings. The Caregiver Report contains three subtests: Problem Behavior (10 items), Personal Self-Care (13 items), and Adaptive Behavior (15 items). All items are rated on a 3-point scale with “Fail” = 0, “Emerge” = 1, and “Pass” = 2. Previous studies have shown that the Cantonese Chinese version of the PEP-3 has good psychometric properties and Cronbach’s alpha coefficients for all subscales were above 0.80 (Shek and Yu 2015).

In addition to the sCPEP-3, the following instruments were used in the present study to examine the convergent validity of the questionnaire.

#### Gesell Developmental Observation-Revised (GDO-R)

The GDO-R (Sivan 2010) is a multidimensional assessment adapted from the original GESELL developmental schedules (Gesell 1940). The questionnaire measures the development of infants and young children in adaptive area, gross motor, fine motor, language area and personal and social area (Sivan 2010). The scores provide professionals with information about a child’s development in relation to the typical growth patterns of children between 2.5 and 9 years of age. A higher score represents a higher level of development. In the present study, it was hypothesized that the GDO-R would be positively correlated with the sCPEP-3.

#### Vineland Adaptive Behavior Scale (VABS)

The VABS (Freeman et al. 1999) is a caregiver-reported measure widely used in different cultures that assesses adaptive behavioral skills in children and adolescents (Gillham et al. 2000; Sparrow et al. 1984). The questionnaire measures five major domains, including communication, daily living skills, socialization, motor skills, and maladaptive behavior. The VABS has been translated into Chinese and validated in different Chinese populations with good psychometric properties (Wu et al. 2004). In the present study, participants’ scores on the VABS subscales were hypothesized to be positively correlated with sCPEP-3 subtest scores.

#### Childhood Autism Rating Scale (CARS)

As one of the most widely used diagnostic instruments, the CARS is a behavioral rating scale that assists professionals in diagnosing children with ASD (Schopler et al. 1980, 1988). It covers 15 different areas of functioning that are crucial for autism: relating to people; imitation; emotional response; body use; object use; adaptation to change; visual response; listening response; taste, smell, and touch response and use; fear or nervousness; verbal communication; nonverbal communication; activity level; level and consistency of intellectual response; and general impressions. Children’s behaviors are observed and rated on a 4-point scale with clear behavioral anchors. The higher the scores on the CARS, the more maladaptive the subjects are when they’re being observed. Unlike the GDO-R and the VABS, which are employed to evaluate the developmental functioning of normal children, the CARS is designed to assess children with special needs and to differentiate children with ASD from other developmental disorders, such as mental retardation. In the current study, it was expected that the CARS subscales and sCPEP-3 subtests would be negatively correlated with each other.

## Results

### Reliability of the sCPEP-3

Internal consistency, test–retest reliability, and inter-rater reliability were examined to provide evidence of the reliability of the sCPEP-3 subtests and composites for the current samples of Chinese children. The results of the three reliability tests are presented and discussed below.

### Internal Consistency

Internal consistency reliability was examined for the subtests and composites of the sCPEP-3, based on the ASD sample at six age intervals. Table 2 first presents the Cronbach’s alpha coefficients and mean inter-item correlation coefficients of the scales. The Cronbach’s alphas ranged from acceptable to good (median = 0.89) for children in different age groups. Nevertheless, the alpha of characteristic verbal behavior (CVB) in age group 2 (2.0–2.9 years) is relatively low (0.59), which may be due to the inability of nearly half of the children in this age group to complete this subtest. For the whole sample of children with ASD, including all age groups, the Cronbach’s alpha coefficients ranged from 0.77 to 0.96 for the Performance subtests, from 0.80 to 0.86 for the Caregiver Report subtests, and from 0.94 to 0.98 for the composites. Mean inter-item correlation coefficients equaled to or exceeded 0.29 for the Performance subtests; equaled to or exceeded 0.29 for the Caregiver Report subtests; and equaled to or exceeded 0.28 for the composites. In general, the high coefficients suggested that the sCPEP-3 subtests and composites had good internal consistency when applied to children with ASD in mainland China.

**Table 2:** Internal consistency reliability of all sCPEP-3 subtests, and test–retest reliability (N = 62) and interrater reliability of the sCPEP-3 Performance Test (N = 59)

sCPEP-3 subtests	Internal consistency reliability									
	Cronbach’s alphas of different age groups							MICC	N	No. of items
	2	3	4	5	6	7	Total			
Performance Test										
CVP	0.93	0.93	0.95	0.95	0.97	0.97	0.95	0.44	549	34
EL	0.95	0.95	0.95	0.94	0.95	0.96	0.95	0.47	551	25
RL	0.95	0.96	0.96	0.95	0.96	0.97	0.96	0.62	553	19
FM	0.85	0.85	0.90	0.86	0.91	0.93	0.89	0.32	554	20
GM	0.85	0.86	0.86	0.63	0.84	0.84	0.81	0.35	554	15
VMI	0.84	0.89	0.89	0.88	0.90	0.91	0.89	0.51	551	10
AE	0.81	0.86	0.86	0.86	0.88	0.93	0.87	0.39	554	11
SR	0.84	0.88	0.88	0.87	0.89	0.92	0.88	0.43	553	12
CMB	0.83	0.86	0.87	0.86	0.90	0.86	0.86	0.30	554	15
CVB	0.59	0.76	0.79	0.84	0.88	0.78	0.77	0.29	496	11
Caregiver Report										
PB	0.75	0.81	0.83	0.83	0.82	0.80	0.81	0.30	551	10
PSC	0.77	0.77	0.84	0.79	0.84	0.81	0.80	0.33	551	13
AB	0.87	0.86	0.85	0.85	0.89	0.85	0.86	0.29	549	15
Composites										
Communication	0.98	0.98	0.98	0.98	0.99	0.99	0.98	0.47	546	78
Motor	0.94	0.94	0.95	0.93	0.96	0.97	0.95	0.49	551	45
Maladaptive behaviors	0.93	0.94	0.94	0.95	0.96	0.93	0.94	0.28	495	49

sCPEP-3 Subtests	Test–retest reliability				*r*	Interrater reliability			
	First testing (N = 62)		Second testing (N = 62)			Polychoric correlation coefficients (N = 59)			
	*M*	*SD*	*M*	*SD*		Min	Max	Median	Mean
Performance Test									
CVP	34.05	15.59	38.22	14.72	0.96^**^	0.36	0.98	0.87	0.81
EL	17.70	13.13	19.52	13.60	0.98^**^	0.32	1.00	0.93	0.88
RL	18.13	11.93	20.75	11.18	0.95^**^	0.61	0.98	0.88	0.87
FM	28.77	7.15	30.43	6.57	0.94^**^	0.47	1.00	0.84	0.81
GM	25.02	5.75	26.46	3.95	0.82^**^	0.20	1.00	0.78	0.78
VMI	12.07	4.85	13.46	4.87	0.90^**^	0.54	0.87	0.71	0.73
AE	14.85	3.56	15.15	3.34	0.78^**^	0.54	0.88	0.70	0.71
SR	13.64	4.12	14.30	4.06	0.89^**^	0.19	0.90	0.65	0.65
CMB	22.87	4.86	22.66	4.29	0.73^**^	0.47	0.87	0.70	0.67
CVB	12.84	3.62	13.61	2.89	0.75^**^	0.33	0.83	0.65	0.64
Caregiver Report									
PB	–	–	–	–	–	–	–	–	–
PSC	–	–	–	–	–	–	–	–	–
AB	–	–	–	–	–	–	–	–	–
Composites									
Communication	–	–	–	–	–	–	–	–	–
Motor	–	–	–	–	–	–	–	–	–
Maladaptive behaviors	–	–	–	–	–	–	–	–	–

*MICC* mean inter-item correlation coefficients, *CVP* cognitive verbal/preverbal, *EL* expressive language, *RL* receptive language, *FM* fine motor, *GM* gross motor, *VMI* visual-motor imitation, *AE* affective expression, *SR* social reciprocity, *CMB* characteristic motor behaviors, *CVB* characteristic verbal behaviors, *PB* problem behaviors, *PSC* personal self-care, *AB* adaptive behavior

**p < .01

### Test–Retest Reliability

The means, standard deviations, and correlation coefficients of the sCPEP-3 subtest scores of participants between two time points are also presented in Table 2. All test–retest correlation coefficients ranged from 0.73 to 0.98, indicating the good time sampling reliability of the sCPEP-3 based on the mainland Chinese sample. This reveals that participants’ performance on the sCPEP-3 is stable, regardless of random factors in the conditions of the participants themselves or the testing environment over time.

### Inter-Rater Reliability

Table 2 summarizes the correlation coefficients for the scores of ten Performance subtests, as rated by two independent raters. Polychoric correlation coefficients were computed for each pair of items, based on the agreement of the two raters, which was used as an indicator of inter-rater reliability (Jöreskog and Sörbom 1988). The skewness of items was calculated to acquire the bivariate normality of data for the polychoric correlation. The results revealed that 76 out of 344 items skewed greater than the absolute value of 2. Among these items, seven items (items 3, 4, 26, 42, 97, 120, and 124) yielded polychoric correlation coefficients that appeared to be significantly biased under the condition of bivariate normality. These correlation coefficients were then excluded in the calculation of the range, mean, and median of the polychoric correlation coefficients for their corresponding subtests. The mean and median polychoric correlation coefficients for each Performance subtest ranged from 0.64 to 0.93, suggesting moderate to very large correlations (Hopkins and Stanley 1981). This provides support for the inter-rater reliability of the sCPEP-3 Performance subtests. The findings suggest that different examiners rated children with ASD using the sCPEP-3 items in a consistent way.

#### Validity of sCPEP-3

Several methods were used to examine the validity of the sCPEP-3. First, to provide evidence of the convergent validity of the instrument, correlation coefficients between participants’ scores on the sCPEP-3 subtests and three other questionnaires measuring similar developmental skills and adaptive behaviors were calculated. Second, to provide evidence of the construct validity of the instrument, participants’ sCPEP-3 subtest scores were compared between the ASD group and the control group, and between male and female children in the ASD group using MANOVA. MANOVA was utilized because multiple dependent variables were involved. Typically developing children in the control group were expected to have better performance than children in the ASD group, while no significant gender difference was identified among children with ASD. Third, the factorial validity of the Performance Test was examined by conducting a confirmatory factor analysis (CFA) in which the ten subtests of the Performance Test (observed variables) were hypothesized to load on the three sCPEP-3 composites (latent constructs), according to the original theoretical model of the questionnaire.

### Convergent Validity

To provide a descriptive profile of the participants’ performance in regard to the three criterion measures, the means and standard deviations of the GDO-R, VABS, and CARS subscales were calculated and are summarized in the supplementary table (Table A). Pearson correlation coefficients between the GDO-R and sCPEP-3 subscale scores were first computed. As can be seen in Table 3, while the subscale of Characteristic Verbal Behaviors in the Performance Test in the sCPEP-3 was not significantly correlated with gross motor and fine motor skills in the GDO-R, this Performance Test subscale was significantly correlated with the other three subscales in GDO-R, with coefficients ranging from 0.33 to 0.51. In addition, all other subscales of the Performance Test and the Caregiver Report in the sCPEP-3 were positively correlated with subscales in the GDO-R, with moderate to high correlation coefficients ranging from 0.28 to 0.82.

**Table 3:** Correlation coefficients between the subscales of the GDO-R, VABS, and CARS, and the sCPEP-3 subtests (N = 60)

	Performance Test										Caregiver Report		
	CVP	EL	RL	FM	GM	VMI	AE	SR	CMB	CVB	PB	PSC	AB
GDO-R													
Adaptive area	0.72**	0.71**	0.62**	0.67**	0.52**	0.62**	0.43**	0.55**	0.54**	0.36*	0.47**	0.35**	0.59**
Gross motor	0.53**	0.50**	0.54**	0.49**	0.47**	0.53**	0.28*	0.42**	0.29*	0.04	0.48**	0.34*	0.48**
Fine motor	0.73**	0.67**	0.60**	0.69**	0.40**	0.60**	0.39**	0.51**	0.57**	0.25	0.36**	0.40**	0.54**
Language area	0.75**	0.82**	0.80**	0.59**	0.57**	0.58**	0.42**	0.65**	0.51**	0.51**	0.44**	0.36**	0.48**
Personal and social area	0.73**	0.72**	0.70**	0.67**	0.57**	0.63**	0.39**	0.58**	0.45**	0.33*	0.35**	0.59**	0.59**
VABS													
Receptive	0.70**	0.77**	0.78**	0.66**	0.54**	0.57**	0.46**	0.70**	0.47**	0.45**	0.29*	0.42**	0.42**
Expressive	0.76**	0.84**	0.82**	0.64**	0.54**	0.55**	0.36**	0.65**	0.46**	0.51**	0.34*	0.42**	0.39**
Written	0.61**	0.70**	0.61**	0.53**	0.35**	0.41**	0.29*	0.46**	0.32*	0.32*	0.25	0.26	0.21
Communication	0.78**	0.87**	0.84**	0.68**	0.55**	0.57**	0.40**	0.68**	0.48**	0.50**	0.34*	0.42**	0.39**
Personal	0.24	0.32*	0.29*	0.16	0.31*	0.12	0.19	0.25	0.14	0.31*	0.09	0.16	0.10
Domestic	0.24	0.29*	0.33*	0.30*	0.02	0.23	0.15	0.32*	0.16	0.12	− 0.11	0.06	− 0.05
Community	0.62**	0.68**	0.64**	0.56**	0.41**	0.46**	0.30*	0.49**	0.36**	0.24	0.12	0.30*	0.12
Skills	0.69**	0.72**	0.70**	0.63**	0.53**	0.52**	0.35**	0.56**	0.40**	0.30*	0.12	0.35**	0.18
Interpersonal relationships	0.53**	0.56**	0.63**	0.49**	0.47**	0.47**	0.37**	0.56**	0.34*	0.23	0.26	0.37**	0.38**
Play and leisure time	0.56**	0.58**	0.60**	0.59**	0.54**	0.59**	0.56**	0.66**	0.46**	0.46**	0.18	0.40**	0.34*
Coping skills	0.29*	0.35**	0.37**	0.30*	0.28*	0.37**	0.34*	0.44**	0.33*	0.24	0.10	0.04	0.06
Socialization	0.55**	0.58**	0.63**	0.54**	0.50**	0.55**	0.48**	0.64**	0.42**	0.35*	0.22	0.34*	0.33*
Gross motor	0.69**	0.67**	0.70**	0.68**	0.56**	0.53**	0.35**	0.53**	0.34*	0.16	0.14	0.44**	0.32*
Fine motor	0.77**	0.77**	0.72**	0.68**	0.47**	0.49**	0.34*	0.56**	0.44**	0.23	0.27*	0.40**	0.31*
Motor skills	0.75**	0.74**	0.73**	0.69**	0.55**	0.54**	0.33*	0.57**	0.39**	0.20	0.17	0.44**	0.32*
Adaptive behavior	0.77**	0.82**	0.81**	0.69**	0.57**	0.58**	0.42**	0.67**	0.49**	0.40**	0.26	0.41**	0.32*
CARS													
Relating to People	− 0.46**	− 0.50**	− 0.51**	− 0.40**	− 0.46**	− 0.38**	− 0.29*	− 0.49**	− 0.27*	− 0.19	− 0.39**	− 0.46**	− 0.46**
Imitation	− 0.55**	− 0.50**	− 0.42**	− 0.47**	− 0.51**	− 0.44**	− 0.26	− 0.46**	− 0.30*	− 0.16	− 0.33*	− 0.49**	− 0.50**
Emotional response	− 0.27	− 0.21	− 0.21	− 0.22	− 0.33*	− 0.29*	− 0.28*	− 0.29*	− 0.19	− 0.25	− 0.42**	− 0.28*	− 0.50**
Body use	− 0.28*	− 0.22	− 0.25	− 0.31*	− 0.43**	− 0.19	− 0.27	− 0.37**	− 0.34*	− 0.11	− 0.31*	− 0.26	− 0.54**
Object use	− 0.36**	− 0.38**	− 0.36**	− 0.37**	− 0.34*	− 0.31*	− 0.30*	− 0.52**	− 0.21	− 0.36*	− 0.20	− 0.23	− 0.31*
Adaptation to change	− 0.22	− 0.16	− 0.20	− 0.17	− 0.20	− 0.10	− 0.18	− 0.20	− 0.12	− 0.01	− 0.25	− 0.12	− 0.26
Visual response	− 0.42**	− 0.41**	− 0.35**	− 0.36**	− 0.36**	− 0.45**	− 0.24	− 0.40**	− 0.24	− 0.21	− 0.40**	− 0.39**	− 0.52**
Listening response	0.02	0.02	0.07	− 0.01	− 0.09	0.08	− 0.14	− 0.19	− 0.19	− 0.29	0.06	0.08	− 0.10
Taste, smell, touch response and use	− 0.32*	− 0.29*	− 0.19	− 0.31*	− 0.35**	− 0.40**	− 0.39**	− 0.36**	− 0.38**	− 0.30*	− 0.17	− 0.27*	− 0.33*
Fear or nervousness	− 0.07	0.01	0.09	− 0.09	− 0.12	− 0.13	− 0.12	0.13	− 0.01	− 0.04	− 0.03	− 0.17	− 0.17
Verbal communication	− 0.52**	− 0.58**	− 0.57**	− 0.40**	− 0.47**	− 0.40**	− 0.42**	− 0.56**	− 0.44**	− 0.39**	− 0.35**	− 0.46**	− 0.53**
Nonverbal communication	− 0.44**	− 0.50**	− 0.48**	− 0.33*	− 0.39**	− 0.32*	− 0.34*	− 0.48**	− 0.31*	− 0.26	− 0.34*	− 0.30*	− 0.41**
Activity level	− 0.21	− 0.19	− 0.17	− 0.29*	− 0.13	− 0.27*	− 0.17	− 0.31*	− 0.21	− 0.27	− 0.05	− 0.10	− 0.16
Level and consistency of intellectual response	− 0.65**	− 0.67**	− 0.61**	− 0.57**	− 0.49**	− 0.57**	− 0.39**	− 0.52**	− 0.38**	− 0.37*	− 0.44**	− 0.41**	− 0.57**
General impressions	− 0.54**	− 0.53**	− 0.53**	− 0.51**	− 0.52**	− 0.52**	− 0.36**	− 0.51**	− 0.41**	− 0.41**	− 0.40**	− 0.41**	− 0.57**

*CVP* Cognitive Verbal/Preverbal, *EL* Expressive Language, *RL* Receptive Language, *FM* Fine Motor, *GM* Gross Motor, *VMI* Visual-Motor Imitation, *AE* Affective Expression, *SR* Social Reciprocity, *CMB* Characteristic Motor Behaviors, *CVB* Characteristic Verbal Behaviors, *PB* Problem Behaviors, *PSC* Personal Self-Care, *AB* Adaptive Behavior

*p < .05, **p < .01

Table 3 also reports the correlation coefficients between the VABS and sCPEP-3 scores. Similarly, the CPEP-3 subtest scores were significantly and positively correlated with most of the VABS subscale scores. For example, although the subscale score of Characteristic Verbal Behaviors in the Performance Test was significantly correlated with nine out of 16 VABS subscale scores, scores for Expressive Language and Receptive Language in the Performance Test were significantly and positively correlated with all VABS subscale scores, with coefficients ranging from 0.29 to 0.87. Besides, scores for the other seven subscales in the Performance Test (i.e., Cognitive Verbal/Preverbal, Fine Motor, Gross Motor, Visual-Motor Imitation, Affective Expression, Social Reciprocity, and Characteristic Motor Behaviors) were positively associated with 14 or 15 out of 16 subscales in the VABS. While the score for the Problem Behaviors subscale in the Caregiver Report of the sCPEP-3 was significantly correlated with four out of 16 subscales of the VABS, scores for the other two subscales in the Caregiver Report were significantly correlated with eight subscales of the VABS.

Correlation coefficients between the 15 subscales of the CARS and sCPEP-3 subtests were computed and are presented in Table 3. The scores of three CARS subscales (i.e., adaptation to change, listening response, fear or nervousness) were not significantly associated with the sCPEP-3 subtest scores. One CARS subscale score (i.e., activity level) was significantly correlated with only three out of ten Performance Test subscales and was not significantly correlated with Caregiver Report subscale scores. However, the scores of the other 11 CARS subscales were negatively correlated with all or most of the sCPEP-3 subtest scores. This finding is similar to previous studies based on the Hong Kong sample (Shek and Yu 2015).

Generally speaking, the above findings indicate that the sCPEP-3 has good convergent validity.

### Construct Validity

The sCPEP-3 was developed with the ability to distinguish typically developing children from autistic children; therefore, there should be significant differences between the scores of typically developing children and autistic children. The MANOVA results are summarized in Table 4. When interpreting the results, Bonferroni’s correction for multiplicity was adopted to adjust the significant level as 0.005 (0.05/10), given that 10 dependent variables were included in the analyses. It should be noted that no Caregiver Report data were collected from the control group in the present study; thus, group differences in the three subscales of the Caregiver Report cannot be examined. The multivariate effect of group was significant for the ten Performance subtests as a group: *F* (10, 781) = 107.75, Wilks’ Lambda = 0.42, *p* < .001. The result of the univariate analyses showed that the mean scores of the children in the control group were significantly higher than those of the children in the ASD group.

**Table 4:** Comparison between the ASD group and the control group using MANOVA, and gender differences in the sCPEP-3 within the ASD group using MANOVA

	ASD Group		Control group		*F*	*p*	Female		Male		*F*	*p*
	Mean	SD	Mean	SD			Mean	SD	Mean	SD		
Performance Test												
CVP	45.23	14.99	56.23	10.07	128.32	< 0.001	44.52	14.93	45.46	14.96	0.26	0.61
EL	26.47	12.96	34.65	8.11	97.84	< 0.001	25.52	11.67	26.67	13.19	0.52	0.47
RL	26.05	10.63	34.69	4.52	181.45	< 0.001	25.68	9.90	26.16	10.77	0.13	0.71
FM	32.99	6.29	37.24	3.85	112.75	< 0.001	32.62	6.85	33.10	6.18	0.38	0.54
GM	27.35	3.79	28.78	1.31	40.51	< 0.001	27.14	4.53	27.40	3.62	0.32	0.57
VMI	15.16	4.49	18.52	2.09	149.87	< 0.001	15.05	4.31	15.20	4.54	0.07	0.79
AE	16.67	3.82	21.48	1.20	455.72	< 0.001	16.59	4.10	16.70	3.78	0.05	0.82
SR	16.32	4.64	22.80	1.89	539.78	< 0.001	16.01	4.51	16.41	4.68	0.47	0.49
CMB	24.53	4.76	29.63	0.97	342.93	< 0.001	24.77	4.86	24.45	4.77	0.29	0.59
CVB	14.30	4.17	21.35	1.41	815.56	< 0.001	13.72	4.05	14.44	4.19	1.94	0.16
Caregiver Report												
PB	–	–	–	–	–	–	10.13	3.65	10.45	3.48	0.56	0.46
PSC	–	–	–	–	–	–	17.73	4.62	18.31	4.45	1.10	0.30
AB	–	–	–	–	–	–	19.18	5.29	19.88	5.22	1.19	0.28

*CVP* Cognitive Verbal/Preverbal, *EL* Expressive Language, *RL* Receptive Language, *FM* Fine Motor, *GM* Gross Motor, *VMI* Visual-Motor Imitation, *AE* Affective Expression, *SR* Social Reciprocity, *CMB* Characteristic Motor Behaviors, *CVB* Characteristic Verbal Behaviors, *PB* Problem Behaviors, *PSC* Personal Self-Care, *AB* Adaptive Behavior

MANOVA was also used to examine gender differences in the sCPEP-3 subtests within the ASD group and the results are summarized in Table 4. The significant level of Bonferroni correction was adjusted to 0.004 (0.05/13), since 13 dependent variables were included in the analyses, in order to control the experiment-wise error rate. No significant differences were found between ASD girls and boys in regard to the combined dependent variables: *F* (13, 466) = 0.72, Wilks’ Lambda = 0.98, *p* = .74. As shown in Table 4, gender effects were not significant among any of the subtests; boys and girls with ASD displayed similar levels of performance across all subtests. These findings are consistent with our expectations and provide support for the validity of the sCPEP-3.

### Factorial Validity

Based on the original theory, it was hypothesized that the ten Performance subtests load on three factors (i.e., the three composites) as follows: CVP, EL, and RL load on Communication; FM, GM, and VMI load on Motor; and AE, SR, CMB, and CVB load on Maladaptive Behavior. The three composites, Communication, Motor, and Maladaptive Behavior, measure different aspects of development and behavior; therefore, they were allowed to be correlated with each other. Using Amos 17.0, the raw scores of 554 children with ASD for the ten Performance subtests were subjected to confirmatory factor analysis (CFA) using the maximum likelihood method. Five indexes of model fit, including the comparative fit index (CFI), the Tucker-Lewis index of fit (TLI), normed fit index (NFI), root mean square error of approximation (RMSEA), and standardized root mean square residual (SRMR), were calculated to evaluate how well the model fits the sample data.

**Fig. 1:**
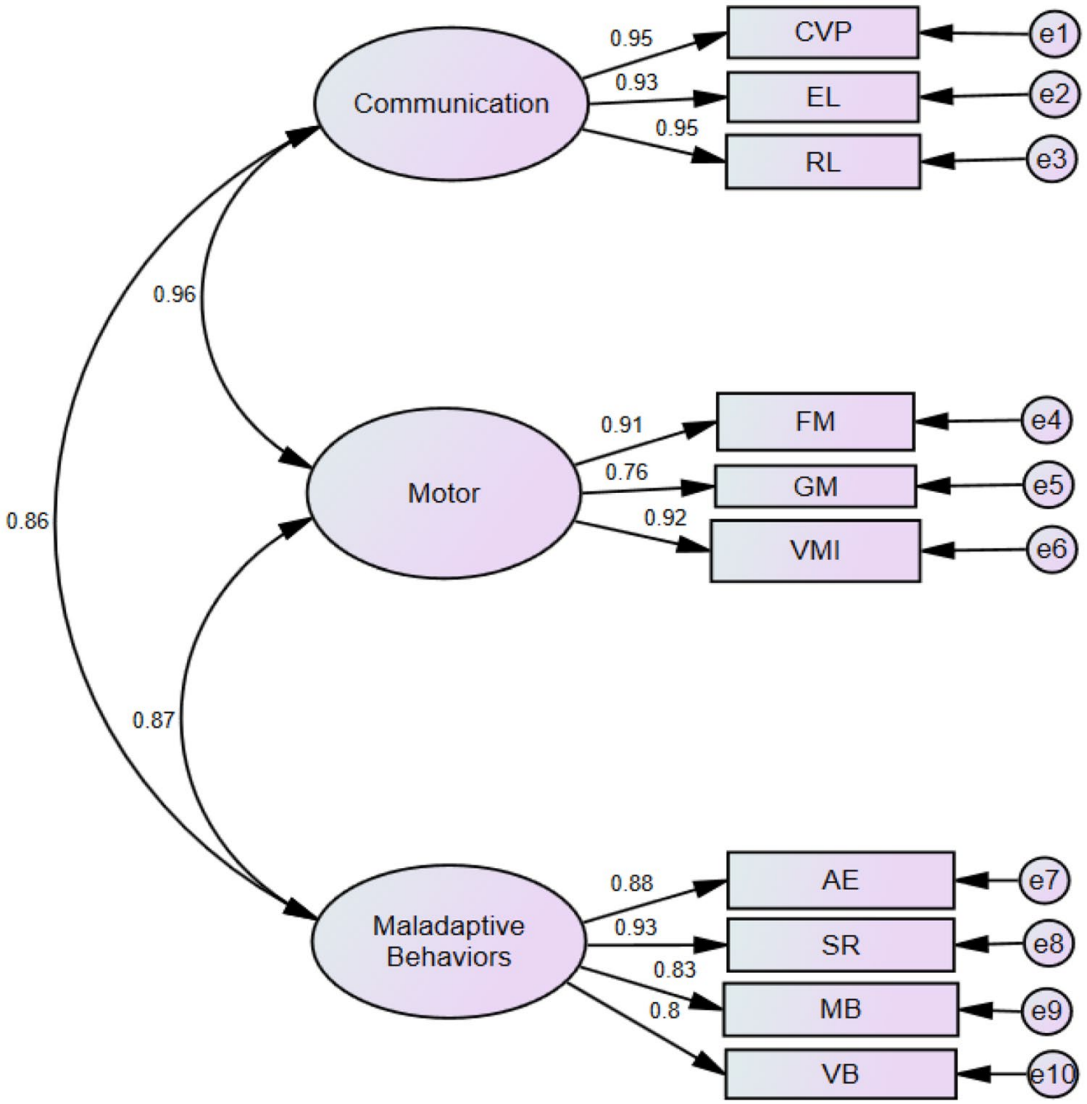
Confirmatory factor analysis of the sCPEP-3 performance tests

Figure 1 shows the results of the CFA of the three-factor model. The ovals in the figure represent the three composites, which are communication, motor, and maladaptive behaviors, respectively. Factor loadings are represented by the values on the arrows between the ovals and rectangles that represent different subtests. These values indicate the influence of the three composites in regard to their corresponding subtests. As presented in the figure, moderate to large sizes of factor loading were observed.

For the model fix indexes, we followed the same criteria for acceptable model fits (i.e., CFI, TLI, and NFI over 0.90 and RMSEA less than 0.10) adopted by the PEP-3 developers (Schopler et al. 2005) and previous researchers (Shek and Yu 2014). The SRMR was used as an extra index in this study, due to its sensitivity to structural model misspecification (Hu and Bentler 1995). A value of less than 0.08 is generally considered to be a good fit. In the present study, *χ*^*2*^_(32)_ = 426.67, CFI = 0.93, TLI = 0.90, NFI = 0.93, SRMR = .03, RMSEA = 0.16. These results indicate a satisfactory fit of the model with the current data and are comparable to the findings reported by the PEP-3 developers, based on American samples (Schopler et al. 2005), and the validation findings using Hong Kong samples (Shek and Yu 2015). Thus, the factorial validity of the Performance Test of the sCPEP-3 is supported.

## Discussion

The present study validated the simplified Chinese version of the Psychoeducational Profile 3rd edition (sCPEP-3) for the assessment of the developmental level of children with ASD in mainland China. Psychometric evaluation of this instrument demonstrated satisfactory internal consistency, test–retest reliability, inter-rater reliability, convergent validity, construct validity, and factorial validity for the current sample of Chinese children. In line with previous studies based on other populations, our findings suggest that, with cultural and linguistic adaptations from the Cantonese Chinese version of the PEP-3 (CPEP-3), the sCPEP-3 is a promising tool that can be used by professionals in mainland China to chart the development of children with ASD in a comprehensive manner. Given that there is a paucity of validated assessment tools in mainland China specifically designed for educational program planning for children with ASD, the present study makes a unique contribution to this field by validating all subtests of both the Performance Test and the Caregiver Report in the sCPEP-3, which can help identify children with ASD both earlier and reliably in mainland China.

In the present study, both the Performance Test and the Caregiver Report of the sCPEP-3 show good internal consistency. Cronbach’s alphas for all subtests and composites are above 0.77, suggesting the homogeneity of all the translated simplified Chinese items in each subscale and composite. However, it is noteworthy that, for three subtests, Cognitive Verbal/Preverbal (CVP), Expressive Language (EL), and Receptive Language (RL), and two composites (i.e., communication and motor), the Cronbach’s alpha coefficients are 0.95 or higher. Similar findings have been reported by researchers in Taiwan (Fu et al. 2010) and Hong Kong (Shek and Yu 2018), which indicates that some items of these subscales may be redundant. Further factor analyses shall be conducted to examine the factor structure of each subtest and to identify highly correlated items. A short form of the sCPEP-3 that requires less time to rate the children may be developed in the future by removing the redundant items.

Our findings provide sound evidence for the validity of the sCPEP-3. Participants with ASD scored significantly lower than children in the control group on all subtests and no gender differences were identified within the ASD group. The results of the confirmatory factor analyses support the three-dimension (communication, motor, and maladaptive behaviors) theoretical structure of the Performance Test. This is consistent with previous findings based on samples of children in Hong Kong (Shek and Yu 2015) and the United States (Schopler et al. 2005). While the results suggest that the factor structure of the PEP-3 Performance Test is stable across different versions of the questionnaire, another implication is that cultural and linguistic differences must be taken into account in scale adaptation, even when the same language is used.

Consistent with our hypothesis, participants’ sCPEP-3 scores were significantly correlated with their scores for most subscales of the GDO-R, VABS, and CARS in the expected directions. Two domains, the Personal and Domestic subscales in the VABS, had relatively low correlation coefficients with the sCPEP-3, compared to other VABS domains. Since the VABS personal subscale reflects how an individual eats, dresses, and practices personal hygiene and the domestic subscale assesses what household tasks the individual can perform (Sivan 2010), it seems reasonable that these two subscales were less correlated with the sCPEP-3 subtests, which mainly focus on the developmental functioning of children.

The validated sCPEP-3 has multiple practical implications for professionals working with mandarin-speaking children with ASD. First, the use of the instrument can promote the development and provision of individualized educational programs and treatment for these children. While in recent years, a number of ASD screening tools (e.g., the Autism Behaviour Checklist) and diagnostic instruments (e.g., the Childhood Autism Rating Scale) have been introduced and validated for Mandarin-speaking children (Li et al. 2005; Yin et al. 2011; Guo et al. 2011; Gong et al. 2011; Zhang et al. 2011; Zhou et al. 2017), validation studies on educational assessment are very limited in mainland China (Sun et al. 2000). With the sCPEP-3, professionals will be able to obtain detailed data and an overall pattern of a child’s developmental strengths and weaknesses, maladaptive behaviors, learning style, and interests, which can inform individualized educational planning. The detailed data also provide important insights into appropriate developmental expectations of the children involved (Huerta and Lord 2012; Johnson and Myers 2007).

Second, as the sCPEP-3 can be flexibly administered without requiring a certain level of verbal ability, attention skills, and concentration of the participants, it can assist clinicians in identifying ASD cases in young children who have previously been considered to be untestable (Sun et al. 2013; Zhou et al. 2017). Chinese clinicians may use the sCPEP-3 in conjunction with other diagnostic tools to improve the diagnostic accuracy of ASD, which may in turn reduce the rate of unrecognized cases in China. Besides, the sCPEP-3 measures a child’s behavioral characteristics specifically associated with ASD and can be used to differentiate ASD from other types of developmental conditions.

Another advantage of the sCPEP-3 is its inclusion of both a formal Performance Test and a Caregiver Report. It has been recommended that a comprehensive evaluation of children with ASD must be based not only on standardized observation scores from skilled examiners, but also on information provided by caregivers (Huerta and Lord 2012). While standardized ratings help professionals to understand a child’s development as compared to typically developing children, caregivers’ reports offer a broader context with which to understand children’s daily behavior, as well as familial and other environmental factors that may influence the children’s performance. With the use of the sCPEP-3, researchers and clinicians are able to integrate information from different sources and gain a complementary picture of children’s development.

Finally, the sCPEP-3 can serve as an outcome measure to evaluate the treatment effects of different ASD programs in China. To our knowledge, the present study is the first to validate the PEP-3, a well-established educational assessment tool for ASD children, in the context of mainland China. The strengths of this study include a relatively large sample size, the inclusion of a control group of typically-developing age-matched children, and a comprehensive evaluation of the different types of psychometric properties of the sCPEP-3.

Meanwhile, several limitations that may temper the conclusion of the study are acknowledged. First, all participants of the ASD group were recruited from a renowned child development center in a hospital in mainland China. Although the children came from different Chinese cities, this sample may not be representative of children with ASD in a broader context of China and the generalizability of the findings is likely to be affected. Future studies should further examine the psychometric properties of the sCPEP-3 based on participants from multiple geographic areas in China.

Second, while the ASD group and the control group were matched in terms of age and gender, other factors that also influence children’s development and performance were not measured, such as intelligence and other possible concurrent diagnoses, particularly other developmental disorders (e.g., Attention Deficit Hyperactivity Disorder). These factors must be taken into account when setting up the inclusion criterion of participant recruitment in future studies.

Third, the three instruments adopted in the present study for the convergent validity test of the sCPEP-3 were developed decades ago in the West. Although they are still frequently used by practitioners, some of the items may not work with the most recent understanding of ASD and capture more subtle autistic symptoms. This may partially explain the relatively low correlation coefficients between the sCPEP-3 subtests and several subscales of these instruments. More recently developed screening and assessment tools for ASD (e.g., the Autism Spectrum Screening Questionnaire) (Guo et al. 2011) could be used in the future to further test the validity of the sCPEP-3. Finally, the inter-rater reliability and test–retest reliability of the Caregiver Report of the sCPEP-3 were not examined in the present study. Whether or not different caregivers can respond to the questionnaire in a consistent way should be examined in the future.

## Conclusion

Despite the limitations outlined above, the present study provides strong support for the reliability and validity of the sCPEP-3, which can be utilized in mainland China. The validated sCPEP-3 provides Chinese practitioners with a useful tool with which to obtain an early and detailed assessment of children with ASD, which is critical for planning and evaluating therapeutic-educational intervention programs based on children’s current developmental states and their changes in different developmental domains over time.

## Electronic supplementary material

Below is the link to the electronic supplementary material.

Supplementary material 1 (DOCX 20 KB)
