# Supplementary material for: Validation of the Simplified Chinese Psychoeducational Profile Third Edition in Mainland China
Source: J Autism Dev Disord. 2018 Dec 12;49(4):1599–612. doi: 10.1007/s10803-018-3827-z (PMC6450835; doi:10.1007/s10803-018-3827-z)
Supplement: Supplementary file 1 — Supplementary material 1 (DOCX 20 KB) [file 10803_2018_3827_MOESM1_ESM.docx]

Supplementary Table

Table A

*Descriptive statistics of GDO-R, VABS, and CARS subscales (N = 60)*

|  | Scales | *Mean* | *SD* | Range |
| --- | --- | --- | --- | --- |
| GDO-R | Adaptive area | 55.97 | 16.29 | 23-95 |
|  | Gross motor | 65.12 | 14.22 | 34-105 |
|  | Fine motor | 63.50 | 19.80 | 23-106 |
|  | Language area | 42.55 | 17.71 | 13-89 |
|  | Personal, social area | 53.53 | 15.35 | 19-86 |
| VABS | Receptive | 6.72 | 3.98 | 0-17 |
|  | Expressive | 12.57 | 12.27 | 0-48 |
|  | Written | 1.42 | 3.45 | 0-16 |
|  | Communication | 20.70 | 18.21 | 0-77 |
|  | Personal | 27.52 | 15.76 | 0-118 |
|  | Domestic | 1.12 | 2.69 | 0-17 |
|  | Community | 5.83 | 7.59 | 0-37 |
|  | Skills | 32.43 | 18.50 | 0-104 |
|  | Interpersonal relationships | 5.45 | 4.84 | 0-23 |
|  | Play and leisure time | 6.50 | 4.01 | 0-22 |
|  | Socialization | 13.43 | 10.24 | 0-64 |
|  | Gross motor | 19.77 | 5.19 | 0-32 |
|  | Motor skills | 31.10 | 10.42 | 0-56 |
|  | Adaptive behavior | 96.70 | 53.09 | 0-301 |
| CARS | Relating to people | 2.58 | 0.70 | 2-4 |
|  | Imitation | 2.57 | 0.72 | 2-4 |
|  | Emotional response | 2.38 | 0.69 | 1-4 |
|  | Body use | 2.15 | 0.58 | 1-4 |
|  | Object use | 2.58 | 0.83 | 1-4 |
|  | Adaptation to change | 2.22 | 0.61 | 1-4 |
|  | Visual response | 2.25 | 0.65 | 1-4 |
|  | Listening response | 1.75 | 0.70 | 1-3 |
|  | Taste, smell, and touch response and use | 1.65 | 0.78 | 1-4 |
|  | Fear or nervousness | 1.55 | 0.57 | 1-3 |
|  | Verbal communication | 2.97 | 0.74 | 1-4 |
|  | Nonverbal communication | 2.58 | 0.72 | 1-4 |
|  | Activity level | 2.27 | 0.63 | 1-4 |
|  | Level and consistency of intellectual response | 2.58 | 0.77 | 1-4 |
|  | General impressions | 2.75 | 0.70 | 2-4 |
